# Traumatic oculomotor nerve avulsion with subarachnoid hemorrhage identified using magnetic resonance imaging

**DOI:** 10.1093/omcr/omaf070

**Published:** 2025-06-27

**Authors:** Keiichiro Tominaga, Takashi Moriya, Tomohiro Kikuchi, Yuki Kishihara, Hideto Yasuda, Masahiro Kashiura, Shinzato Yutaro

**Affiliations:** Department of Emergency and Critical Care Medicine, Jichi Medical University Saitama Medical Center, 1-847 Amanuma-cho, Omiya-ku, Saitama-shi, Saitama, Japan; Department of Emergency and Critical Care Medicine, Jichi Medical University Saitama Medical Center, 1-847 Amanuma-cho, Omiya-ku, Saitama-shi, Saitama, Japan; Department of Emergency and Critical Care Medicine, Jichi Medical University Saitama Medical Center, 1-847 Amanuma-cho, Omiya-ku, Saitama-shi, Saitama, Japan; Department of Date Science Center, Jichi Medical University, 1-3311 Yakushiji, Shimotsuke-shi, Tochigi, Japan; Department of Emergency and Critical Care Medicine, Jichi Medical University Saitama Medical Center, 1-847 Amanuma-cho, Omiya-ku, Saitama-shi, Saitama, Japan; Department of Emergency and Critical Care Medicine, Jichi Medical University Saitama Medical Center, 1-847 Amanuma-cho, Omiya-ku, Saitama-shi, Saitama, Japan; Department of Clinical Research Education and Training Unit, Keio University Hospital Clinical and Translational Research Center (CTR), 35 Shinanomachi, Shinjuku-ku, Tokyo, Japan; Department of Emergency and Critical Care Medicine, Jichi Medical University Saitama Medical Center, 1-847 Amanuma-cho, Omiya-ku, Saitama-shi, Saitama, Japan; Department of Emergency and Critical Care Medicine, Jichi Medical University Saitama Medical Center, 1-847 Amanuma-cho, Omiya-ku, Saitama-shi, Saitama, Japan

**Keywords:** car accident, case report, MRI CISS, oculomotor nerve avulsion, petroclinoid ligament

## Abstract

Traumatic injury to the oculomotor nerve is a serious condition and generally carries a poor prognosis.Herein, we report a case of oculomotor nerve disruption with traumatic subarachnoid hemorrhage that was identified using an imaging technique. A 63-year-old female patient was brought to our emergency department following a traffic accident. Conservative treatment was initiated, leading to an improvement in her level of consciousness, although her right oculomotor nerve palsy symptoms remained unalleviated. She was discharged 2 weeks later with persistent symptoms of right oculomotor nerve palsy. In cases of cranial nerve palsy following head injuries, magnetic resonance imaging using steady-state constructive interference can provide valuable insights for detecting damage within the tentorial gap.

## Introduction

Traumatic injury to the oculomotor nerve is a serious condition and generally carries a poor prognosis [1]. As the oculomotor nerve plays a critical role in most eye movements, injuries can result in diplopia, which can impair visual function and quality of life. Such injuries are typically caused by vertical shearing forces within the brainstem, arising from anteroposterior impacts to the frontal or occipital lobes. In cases where paralysis persists without improvement, there is often a high incidence of associated traumatic subarachnoid hemorrhage (SAH) and skull fractures [2]. Diagnosing oculomotor nerve injuries is particularly challenging when the damage occurs within the tentorial gap. Traditional imaging techniques such as computed tomography (CT) often fail to visualize these injuries owing to the complex anatomy of the region and limitations of standard imaging resolution [3]. Thus, diagnosis and treatment have historically relied on observing symptom progression, which can lead to delayed intervention. Herein, we report a case of traumatic SAH and oculomotor nerve disruption identified using magnetic resonance imaging (MRI) with the constructive interference in steady state (CISS) technique. This method provides high-resolution visualization of neural structures, allowing for early and precise diagnoses in cases of suspected oculomotor nerve injury. This case highlights the utility of the CISS technique for addressing diagnostic challenges associated with such injuries.

## Case report

A 63-year-old woman was transported to the emergency department after being struck by a car while riding her bicycle, sustaining facial injuries upon collision with the handlebars.

**Figure 1:**
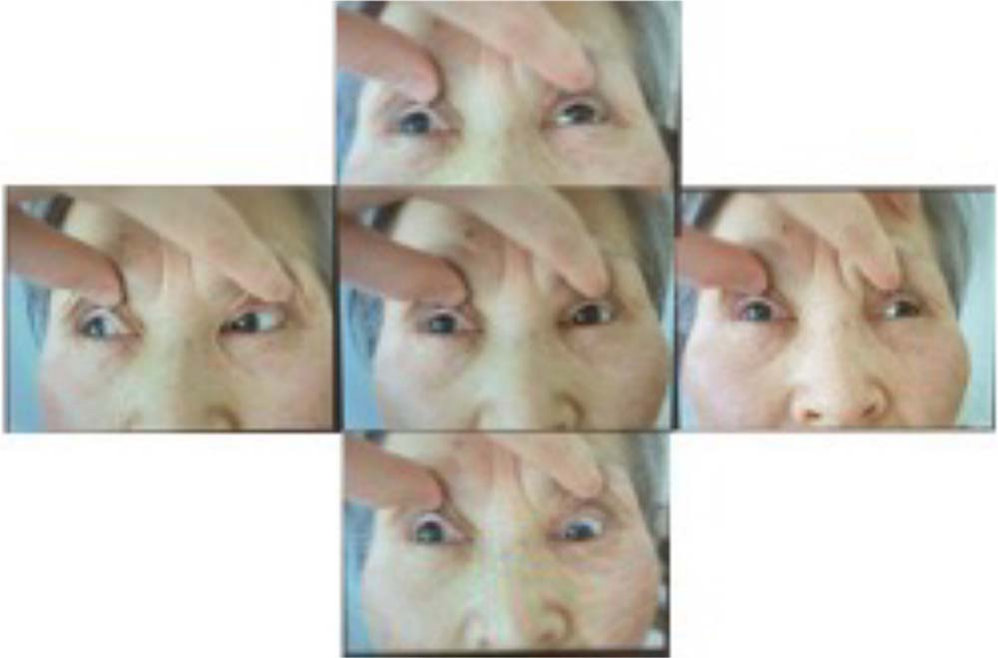
Photographs of the patient upon her arrival to the emergency department, showcasing oculomotor palsy, bilateral ptoses, and abducted eyes.

Her vital signs upon arrival were as follows: temperature, 36.4°C; blood pressure, 145/65 mmHg; and pulse rate, 87 bpm. No traumatic markings were observed on the surface of the head. Her consciousness level was G4V4M6, with signs of confusion such as disorientation regarding her location. She had a drooping right eyelid and impaired movement in her right eye, characterized by an outward deviation and inability to move inward, upward, or downward (Fig. 1). Her pupil size was 8/5.5 mm, indicating anisocoria. Direct and consensual light reflexes were absent in her right eye. No other neurological abnormalities were observed.

**Figure 2:**
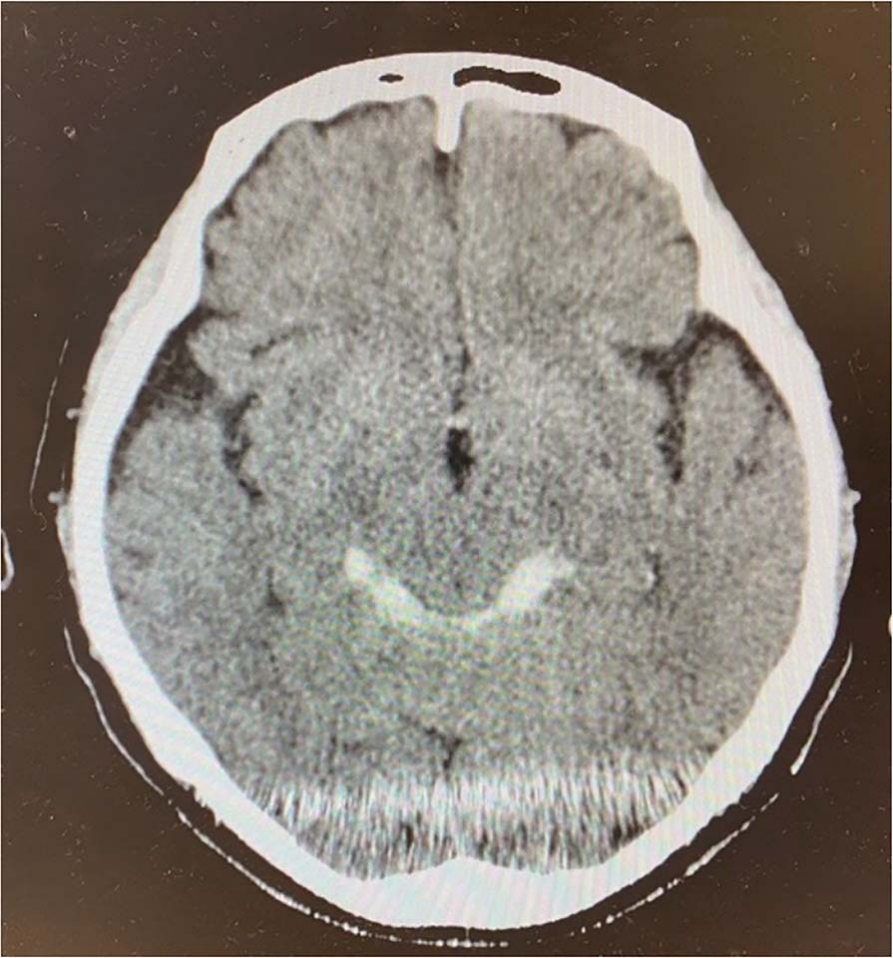
Brain computed tomography on day 3 of the patient’s hospitalization, showing a small high-density area indicating hemorrhage in the bilateral ambient cistern.

**Figure 3:**
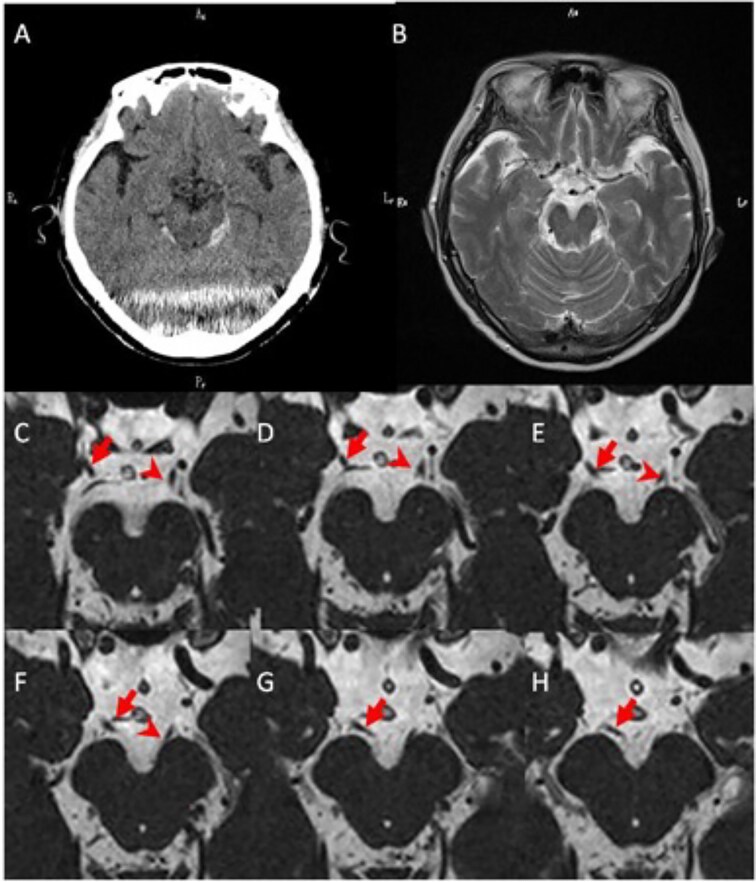
Brain CT on admission and MRI on day 3. (A) A head CT scan showing SAH findings in the bilateral ambient cisterns. (B) MRI T2 images showing no intraparenchymal brain damage. (C–H) Images C–H are MRI CISS images from anterior to posterior. The distal end of the right oculomotor nerve is forming a blind stump (arrow). The left oculomotor nerve is normally connected to the midbrain (arrowhead). CT, computed tomography; MRI, magnetic resonance imaging; SAH, subarachnoid hemorrhage; CISS, constructive interference in steady state.

Head CT showed no fractures but indicated SAH around the brainstem (Fig. 2). No aneurysms were detected on 3D-CT, eliminating an endogenous cause for the SAH. Conservative treatment was initiated, which improved the consciousness level but did not alleviate the symptoms of right oculomotor nerve palsy. On day 3 of hospitalization, a head MRI revealed avulsion of the right oculomotor nerve (Fig. 3) without brain tissue damage. Traumatic injuries occurred only in the brain and oculomotor nerve. As the oculomotor nerve had been severed, symptom improvement could not be expected without surgical intervention. However, the patient declined surgery and was discharged from the hospital 2 weeks later. A follow-up phone call confirmed that the oculomotor nerve palsy remained unchanged 2 years after the accident.

**Figure 4:**
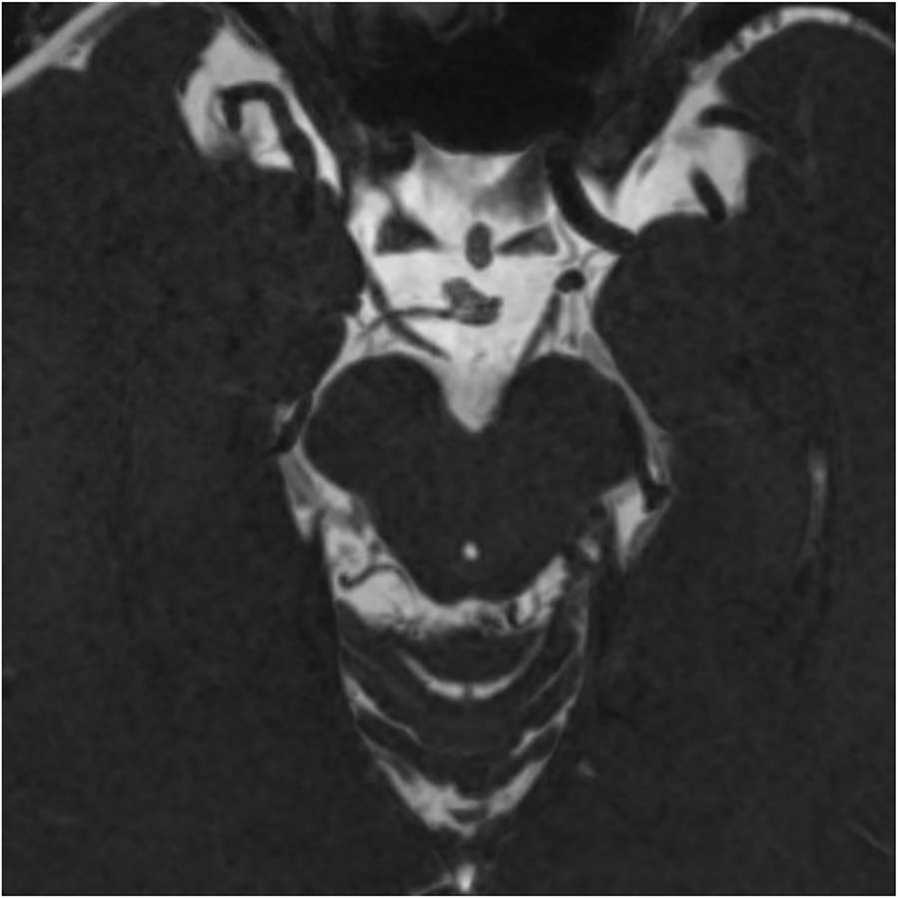
A reconstructed image of Fig. 3 using the minimum intensity projection (MinIP) display method on the CISS imaging for clearer visualization of the nerve stump.

## Discussion

In this case of oculomotor nerve injury with SAH caused by head trauma, we successfully identified avulsion of the oculomotor nerve from the midbrain, using the MRI CISS technique.

The possible causes of injury to the oculomotor nerve leading to palsy following traumatic brain injury include root avulsion at the exit of the nerve from the brainstem, distal fascicular damage, stretching of the nerve (including the parasellar segment), and decreased blood supply [4, 5].

The present case involved root avulsion at the nerve exit from the brainstem. Blunt trauma to the frontal lobe region can generate a linear acceleration force along the rostrocaudal axis, which runs parallel to the oculomotor nerve. This force can cause downward movement of the brainstem, producing shearing forces that stretch and damage the oculomotor nerve, particularly as it crosses the posterior petroclinoid ligament [6].

This mechanism is widely accepted as the cause of complete ONP, even in cases of mild trauma without significant head lesions. Uncal herniation caused by mass effects, wherein the sharp edge of the tentorium compresses and damages the oculomotor nerve, is also linked to ONP [7].

If imaging reveals SAH around the brainstem accompanied by eye movement disorders, it suggests the involvement of these mechanisms. Our case also suggests that root avulsion at the nerve exit from the brainstem occurred due to these forces.

The diagnosis of oculomotor nerve injury requires long-term symptom observation and imaging studies. Outcomes are generally poor, with symptoms such as diplopia potentially impairing quality of life. In cases of complete and irreversible damage, more than 6 months of medication, clinic visits, and symptom monitoring are often necessary [8].

When the extent of the damage cannot be assessed on CT, advanced imaging techniques such as MRI may provide more accurate diagnostic information. However, certain areas, such as the site where the nerve penetrates the dura or posterior petroclinoid ligament within the tentorial notch, remain difficult to evaluate using conventional imaging techniques. To the best of our knowledge, no previous reports have identified such injuries using imaging [9].

The CISS technique offers excellent spatial resolution for distinguishing cerebrospinal fluid from other structures. It is highly effective for visualizing nerves in the brain and spinal cord, making it a valuable tool for identifying nerve damage within the tentorial notch.

In cases of complete oculomotor nerve damage, surgical treatment aims to address functional and cosmetic issues. Surgical interventions include supra-maximal recession and resection of the horizontal rectus muscles, with additional procedures such as horizontal rectus transposition, and globe fixation potentially performed during re-operations [10]. In this case, extraocular muscle surgery was recommended to achieve binocular vision in the patient’s primary gaze but the patient declined surgery for personal reasons. Despite the early detection of nerve rupture, the prognosis was poor because of the severity of the damage, which included complete rupture of the oculomotor nerve at the midbrain.

CISS imaging played a crucial role in precisely identifying our patient’s nerve damage within the tentorial notch, allowing for accurate prognostic discussions and facilitating an understanding of her limited recovery potential (Fig. 3). This case highlights the value of this advanced diagnostic tool when investigating and managing similar injuries.

## Conclusion

Oculomotor nerve injury caused by traumatic brain injury is most commonly observed at the nerve’s dural penetration site or the posterior petroclinoid ligament within the tentorial notch. The CISS technique is a highly effective tool for its early diagnosis, enabling prompt surgical planning for extraocular muscle repair and minimizing the need for long-term care.

In cases with eye movement disorders where imaging reveals SAH around the brainstem, oculomotor nerve injury should be strongly suspected. Further epidemiological research using the CISS technique for traumatic oculomotor nerve injuries is essential to deepen the current understanding of prognostic factors, improve treatment strategies, and explore interventions that may enhance long-term outcomes for patients.

## Supplementary Material

export_file_omaf070
